# Status and Prospects of Cubic Silicon Carbide Power Electronics Device Technology

**DOI:** 10.3390/ma14195831

**Published:** 2021-10-05

**Authors:** Fan Li, Fabrizio Roccaforte, Giuseppe Greco, Patrick Fiorenza, Francesco La Via, Amador Pérez-Tomas, Jonathan Edward Evans, Craig Arthur Fisher, Finn Alec Monaghan, Philip Andrew Mawby, Mike Jennings

**Affiliations:** 1 Newport Wafer Fab, Cardiff Rd, Duffryn, Newport NP10 8YJ, UK; Fan.Li@nptwf.com; 2 Consiglio Nazionale delle Ricerche—Istituto per la Microelettronica e Microsistemi (CNR-IMM), Strada VIII n. 5-Zona Industriale, 95121 Catania, Italy; fabrizio.roccaforte@imm.cnr.it (F.R.); giuseppe.greco@imm.cnr.it (G.G.); patrick.fiorenza@imm.cnr.it (P.F.); francesco.lavia@imm.cnr.it (F.L.V.); 3 Institut Català de Nanociència i Nanotecnologia (ICN2), Universitat Autònoma de Barcelona, 08193 Barcelona, Spain; amador.perez@icn2.cat; 4 Faculty of Science, Bay Campus, College of Engineering, Swansea University, Fabian Way, Crymlyn Burrows, Skewen, Swansea SA1 8EN, UK; j.e.evans@swansea.ac.uk (J.E.E.); c.a.fisher@swansea.ac.uk (C.A.F.); f.a.j.monaghan.909302@swansea.ac.uk (F.A.M.); 5 School of Engineering, The University of Warwick, Gibbet Hill Rd, Coventry CV4 7AL, UK; p.a.mawby@swansea.ac.uk

**Keywords:** 3C-SiC, cubic silicon carbide, power electronics

## Abstract

Wide bandgap (WBG) semiconductors are becoming more widely accepted for use in power electronics due to their superior electrical energy efficiencies and improved power densities. Although WBG cubic silicon carbide (3C-SiC) displays a modest bandgap compared to its commercial counterparts (4H-silicon carbide and gallium nitride), this material has excellent attributes as the WBG semiconductor of choice for low-resistance, reliable diode and MOS devices. At present the material remains firmly in the research domain due to numerous technological impediments that hamper its widespread adoption. The most obvious obstacle is defect-free 3C-SiC; presently, 3C-SiC bulk and heteroepitaxial (on-silicon) display high defect densities such as stacking faults and antiphase boundaries. Moreover, heteroepitaxy 3C-SiC-on-silicon means low temperature processing budgets are imposed upon the system (max. temperature limited to ~1400 °C) limiting selective doping realisation. This paper will give a brief overview of some of the scientific aspects associated with 3C-SiC processing technology in addition to focussing on the latest state of the art results. A particular focus will be placed upon key process steps such as Schottky and ohmic contacts, ion implantation and MOS processing including reliability. Finally, the paper will discuss some device prototypes (diodes and MOSFET) and draw conclusions around the prospects for 3C-SiC devices based upon the processing technology presented.

## 1. Introduction

Power electronics is a key enabling technology for energy generation, transmission, distribution and motion. The importance of this technology is emphasised by the fact that a 40% increase in energy consumption within 20 years is expected [1]. Moreover, 80% of electrical energy will be processed by a power electronic converter by 2030 [2]. Recently, power electronic converter and device technology has been driven by the huge demand seen within the electric vehicle (EV) sector. EV sales are set to reach 18 million by 2023, representing 16.2% of total global vehicle sales [3]. Together, these circumstances project the ever-increasing demand for power electronics on a global scale. In order to meet this required capacity and while still safeguarding our environment, power converters with near-100% energy-efficiency that are lightweight and compact need to be delivered. Furthermore, attention must be paid to the lifetime (or longevity) of these systems, meaning increased reliability within the field.

Such a step-change intervention within the world of power electronics requires advancements within the fundamental semiconductor materials that serve to underpin our energy landscape. The underpinning technologies with respect to power electronics are its constituent high-voltage semiconductor devices. Consequently, these devices represent the largest cost associated with the overall power converter (40% of the total bill of materials for a typical 50 kW EV inverter). Traditionally for the last 50 years, silicon (Si) has dominated the power electronics industry as the semiconductor material of choice. However, the demand for increased energy-efficiency and power density together with higher voltage and current operation mean that a new era in semiconductor materials has dawned. Wide bandgap (WBG) semiconductor materials come with the promise to overcome the inherent material limits imposed by Si. 4H-silicon carbide (4H-SiC) and gallium nitride (2H-GaN or GaN) have emerged as the WBG materials of choice that have replaced Si in many power electronic applications.

For the moment, GaN devices that are based mainly on the high electron mobility transistor (HEMT) architecture are limited commercially to a maximum of 650 V. From the reliability perspective, GaN HEMTs have traditionally suffered from a poor thermal conductivity and the “current collapse” phenomenon, degrading their ability to function within harsh environments and high reliability electronics [4]. 4H-SiC, on the other hand, suffers from numerous reliability issues that are hampering its widespread uptake within the automotive sector. In particular, although SiC Trench MOSFETs exhibit superior on-state resistance compared to both GaN and silicon, the ruggedness of the gate oxide is the limiting factor. Gonzalez et al. [5] note that the competing WBG material technologies centre around the 650 V mark.

Early stage research devices are based on so-called ultrawide bandgap oxide materials such as gallium oxide (Ga_2_O_3_, with β-Ga_2_O_3_ being the most stable). Thus far, β-Ga_2_O_3_ suffers from a poor thermal conductivity, a modest bulk mobility and lack of p-type conductivity. Other ultrawide bandgap materials, including diamond and aluminium nitride (AlN), suffer from a lack of n-type conductivity and a poor bulk electron mobility, respectively. It should be noted that GaN, β-Ga_2_O_3_, and AlN are direct bandgap materials, which severely limits bipolar operation, which is required for higher voltages [6].

This review will place the cubic SiC (3C-SiC) material into the context of power electronic devices; however, it should be noted that other application areas such as biomedical sensors and micro-electromechanical systems (MEMS) are also appropriate and more popular for this SiC polytype. The authors will endeavour to provide a brief insight into some of the advantages of 3C-SiC from the scientific materials perspective in addition to some of the technological issues that must be overcome to realise competitive power MOSFETs and diodes. In particular, the focus will be placed on fundamental semiconductor fabrication technologies; the 3C-SiC/SiO_2_ metal-oxide-semiconductor (MOS) interface, ion implantation, ohmic and Schottky contacts.

## 2. Cubic Silicon Carbide (3C-SiC): Structure and Material Properties for Power Electronic Application

The cubic form of SiC, coined ‘3C-SiC’, is one of many stable polytypes characterised by its wide bandgap and bilayer stacking sequence of ABCABC… [7]. The resulting structure is a pure zinc-blende exhibiting an energy band gap of 2.3–2.4 eV [8], lower compared to other major SiC polytypes, but with a higher electron mobility and saturation velocity owing to its higher degree of symmetry. Although 3C-SiC has a smaller energy bandgap compared to its wide bandgap counterparts such as 4H-SiC and GaN, this material displays isotropy for many of the desired power device material characteristics such as avalanche coefficients and high electron mobility [9,10]. Another advantage of 3C-SiC is its relatively large thermodynamic stability meaning that bulk material can be grown at reduced thermal budgets (below 1500 °C). Table 1 shows the important physical and electrical properties of 3C-SiC compared to other commercial power device materials such as Si, GaN and 4H-SiC. Likewise included are promising oxide and nitride ultra-WBG materials. The 3C-SiC intrinsic carrier concentration (~10^−1^ cm^−3^) is several orders of magnitude lower than in Si, but not as low as 4H-SiC or GaN. Moreover, 3C-SiC has a thermal conductivity three times that of Si. Consequently, 3C-SiC devices should have lower leakage currents with the ability to operate at moderately higher temperatures when compared to Si and GaN. Other key aspects are the reasonable critical electric field value resulting in a higher breakdown of the material. On analysis of these material properties, 3C-SiC is a promising semiconductor for power semiconductor devices in the region of 600–1000 V. On reflection, there exists the possibility to obtain a targeted breakdown voltage (V_B_) with thinner, more highly doped drift layers, which results in a significant reduction of the specific on-resistance (R_ON_) compared to Si devices. Therefore, devices that are smaller and more efficient can be fabricated, minimizing both the static and dynamic losses.

**Figure 1:**
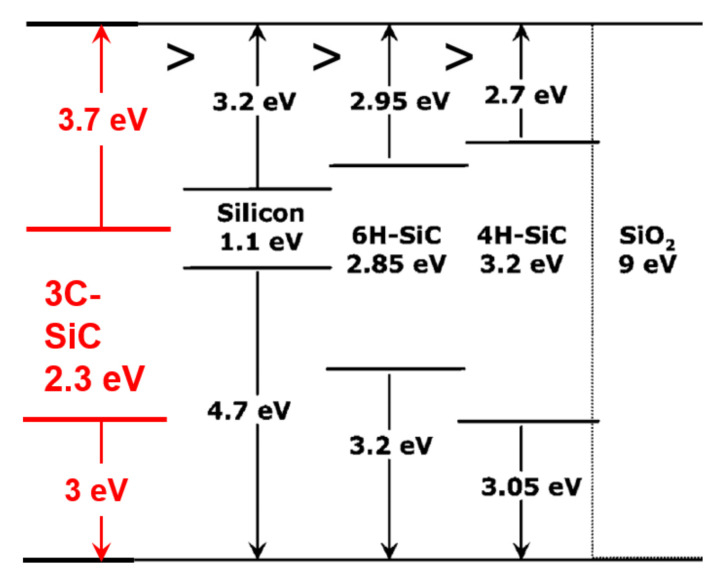
Major power semiconductors’ band structure for 3C-SiC, 4H-SiC, 6H-SiC and silicon, illustrating band offsets with silicon dioxide (SiO_2_).

The 3C-SiC Baliga figure of merit (BFOM) and BFOM for high-frequency, high-power unipolar switches (BHFFOM) [11] are 140 and 25, respectively. These values seem very modest compared to the equivalent values for more advanced WBG power semiconductors such as 4H-SiC and GaN. These key performance indicators for power semiconductors quantify the minimum conduction loss during DC operation (BFOM) and the minimum conduction loss at high frequencies (BHFFOM). Indeed, examination of these values suggests that lower resistance devices are possible based on 4H-SiC and GaN when compared to 3C-SiC. However, this advantage must be weighed against power device reliability and field lifetime within a converter application. In this regard, 3C-SiC is the clear winner, benefitting from a favourable metal-oxide-semiconductor (MOS) interface when compared to its 4H-SiC counterpart. The bandgap value (E_g_) for 3C-SiC was reported by Bimberg et al. [12] and later by Goldberg et al. [8] (see Table 1). Figure 1 shows the conduction band offsets of the major power semiconductors with silicon dioxide (SiO_2_). From the figure it is seen that the band offset (Φ_B_) between 3C-SiC and SiO_2_ is 3.7 eV. This is significantly larger when compared to the other power semiconductors with their values ranging between 2.7 eV–3.2 eV.

The ramifications of this important property are realised in terms of reduced gate leakage current for a given oxide electric field. The important current transport mechanism which relates to this physical parameter is the Fowler-Nordheim (F-N) tunnelling mechanism. The F-N tunnelling current is given by:

(1) $$J_{FN}=\frac{A}{\Phi_{B}}E_{ox}{}^{2}\exp\left(-\frac{B\Phi_{B}{}^{3/2}}{E_{ox}}\right)$$

where *E_ox_* is the oxide electric field, Φ*_B_* is the barrier height and A, B are constant values. It can be seen that due to F-N tunnelling the oxide electric field value must be reduced by 2–3 times in 4H-SiC compared to the 3C-SiC system.

**Figure 2:**
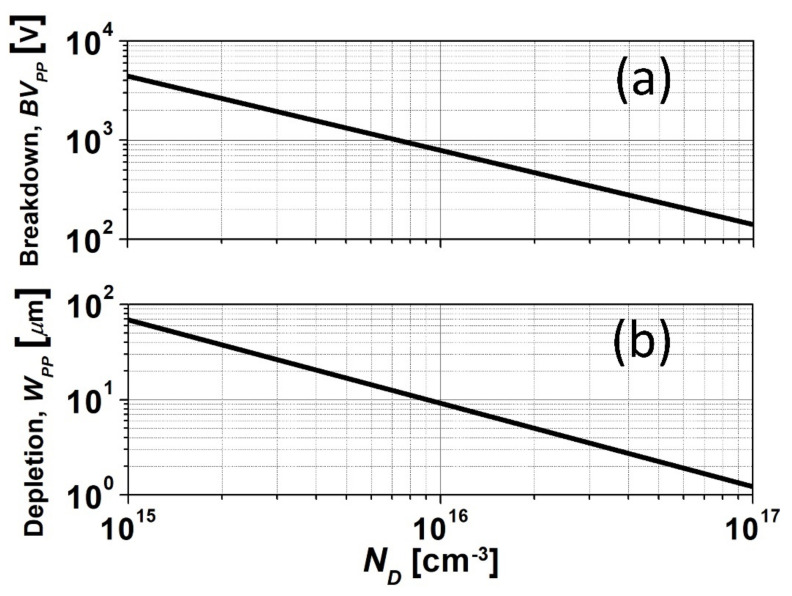
(**a**) Parallel plane breakdown voltage (*BV_PP_*) and (**b**) depletion width (*W_PP_*) as a function of doping (*N_D_*) for 3C-SiC.

Fardi and Van Zeghbroeck [13] developed an empirical breakdown field model based on the breakdown voltage and field values that were obtained from published experimental data [14,15]. This proved to be more than adequate for 3C-SiC device design, having matched electrical breakdown characteristics to many published reports. Moreover, the model has been utilised in commercial 2-dimensional device design suites [16,17,18]. Fitting these impact ionisation coefficients to the electric field and substituting into the impact ionisation integral leads to closed-form solutions of the breakdown voltage and depletion layer width. These material parameters allow for the initial stages of power device design. The closed-form solutions for the breakdown voltage and parallel-plane depletion region width are given as:

(2) $$BV_{PP}=7.88\times 10^{14}N_{D}{}^{-3/4}\;$$

(3) $$W_{PP}=9.12\times 10^{10}N_{D}{}^{-7/8}\;$$

where *BV_PP_* is the breakdown voltage, *N_D_* is the doping concentration and *W_PP_* is the parallel-plane depletion region width. The breakdown voltage and depletion region widths predicted by Equations (2) and (3), respectively, are shown in Figure 2.

## 3. Processing Technology for 3C-SiC

### 3.1. Schottky Contact

One of the main challenges in the processing of electronic devices based on 3C-SiC is the achievement of good quality rectifying contacts, i.e., with almost ideal characteristics and reasonably low leakage current. Several works investigated the properties of Schottky contacts on n-type 3C-SiC over the last three decades. In particular, as summarized in Table 2, most of these works have been performed on 3C-SiC layers grown on Si substrates, using high work-function Schottky contact metals (e.g., Au or Pt). However, the experimental values of the Schottky Barrier Height (SBH), as determined by I-V or C-V measurements, typically lie below 1 eV, i.e., which are much lower than the theoretical predictions of the Schottky–Mott theory.

Eriksson et al. [19] demonstrated the key role of the material quality on the properties of the metal/3C-SiC contacts, showing that double position boundaries (DPB) in 3C-SiC layers grown onto on-axis 4H-SiC can be “killer defects” in large area devices that compromise the functionality of the rectifying barrier [20]. In this work, a novel approach based on Conductive Atomic Force Microscopy (C-AFM) was proposed to characterize Schottky barriers on 3C-SiC in small area devices, establishing a direct relation between the electrical properties of the barrier and the contact area. In particular, reducing the size of the contact resulted in a drastic increase in the measured Au/3C-SiC barrier height, until reaching a value of 1.39 eV for a diode radius of 5 µm, thus demonstrating that the poor rectifying behaviour was due to the high defects density in the material [19].

**Figure 3:**
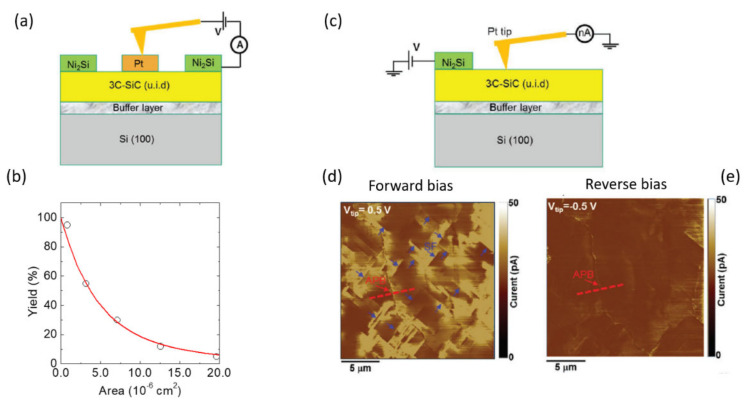
(**a**) Schematic of the C-AFM set-up to probe Pt/3C-SiC Schottky diodes of different areas. (**b**) Percentage of the diodes (yield) with a reverse leakage lower than 10 μA cm^−2^, as a function of diode area. (**c**) Schematic of the C-AFM set-up to probe the 3C-SiC surface and current maps acquired under forward bias (**d**) and reverse bias (**e**). Adapted with permission from Ref. [21]. Copyright © 2021 Wiley VCH.

More recently, using a similar nanoscale approach on 3C-SiC layers grown on Si, Giannazzo et al. [21] confirmed that the device yield, defined as the fraction of diodes with a leakage current lower than 10 μA/cm^2^ (see Figure 3a,b) increases with decreasing the device area. Moreover, this work better clarified the role of specific defects by direct probing of the 3C-SiC surface by C-AFM (see Figure 3c–e). In particular, these measurements showed that antiphase boundaries (APBs) are the main defects responsible for reverse leakage current, while both APBs and stacking faults (SFs) worked as preferential current paths under forward bias of the contact.

**Table 2:** Collection of literature results on Schottky contacts on 3C-SiC materials.

Metal	3C-SiC Orientation	Growing Substrate	Schottky Barrier Height (eV)	Ideality Factor	Extraction Method	Ref.
Au	100	Si	1.15	N.A.	C-V	[22]
Au	100	Si	1.2	1.5	C-V	[23]
Au	111, 100	Si	1.0–1.6	N.A.	C-V	[24]
Pt			1.3–1.8			
Pt	100	Si	0.95 (as dep) −1.35 (800 °C)	N.A.	C-V	[25]
Pd	100	Si	0.92, 0.95	N.A.	C-V, XPS	[26]
Au			0.87, 0.78			
Co			0.73, 0.69			
Au	100	Si	0.47–0.69	1.58–2.30	I-V	[27]
Pd	100	Si	0.42–0.60	3.02–5.28	I-V	[28]
Ti	100	3C-SiC	0.4, N.A.	N.A.	I-V, C-V	[29]
Au			0.67, 0.65			
Ni			0.56, 0.54			
Au	111	4H-SiC	0.7, 1.39	>2	I-V, I-V by C-AFM	[19]
Pt	100	3C-SiC	0.77 (as dep) −1.12 (500 °C)	N.A.		[30]
Au	111	4H-SiC	0.73–0.76	N.A.	I-V by C-AFM	[31]

Clearly, all these results indicate that a significant improvement of the material quality (namely, a reduction of specific defects’ density) remains the only possible route for the achievement of operational Schottky contacts on 3C-SiC materials suitable for power electronics applications.

### 3.2. Ion Implantation and Activation

High impurity doping is necessary for low ohmic contact and sheet resistance in 3C-SiC power devices. The most commonly used dopants for 3C-SiC are nitrogen or phosphorus for n-type, and mainly aluminium for p-type.

The low diffusivity of typical dopants in SiC below 1800 °C [32] means that highly doped selective regions of SiC power devices are often achieved by ion implantation. As implanted dopant species are nearly always interstitial (not chemically bonded), they are therefore electrically inert. Therefore, an extra post implant annealing (PIA) step is typically deployed to repair the lattice damage and place the implanted dopants into their correct substitutional positions. This is referred to as ‘activation’. Extremely high temperatures are required for the SiC PIA; above 1400 °C [33,34] is common for n-type SiC and higher still (>1600 °C) for p-type [35,36,37]. The higher p-type PIA temperature is required because acceptors sit deeper in the band gap than donors, and are consequently more challenging to activate. Regarding 3C-SiC, the most common form is grown heteroepitaxially on Si. As a consequence, these activation annealing temperatures are often limited to 1412 °C (Si melting point). Performing the ion implantation at a higher temperature helps to reduce the induced lattice damage; thus, it is often applied for high dose implantations. Since the ion implantation induced lattice damage increases with the number of dopants per unit volume (namely the dose), hot implants are almost mandatory when the implant concentration goes above 10^19^ cm^−3^ [38].

High temperature PIA also causes a rough semiconductor surface, which is enhanced within implanted regions and can degrade the performance of critical interfaces such as Schottky contacts and MOSFET channels [39,40,41]. A graphite capping layer, demonstrated to be effective up to 1800 °C [35], is often utilised to protect the SiC surface during the PIA and reduce the resulting roughness. Comparing the few examples in the literature, n-type implanted 3C-SiC have been extensively studied for varying annealing conditions (1150 °C to 1400 °C) both with a graphite capping layer [42] and without [43,44]. It was shown that there was little advantage demonstrated when using a graphite cap, likely due to the annealing temperature (below 1400 °C due to the Si substrate) not being high enough to roughen the surface. In [45], it was shown that by combing the use of hot implant and pulsed excimer laser processing, which only anneals the surface region, 3C-SiC crystal damage due to implantation can be effectively repaired without degrading the surface morphology (energy density 0.2444 J/cm^2^ at 10 Hz), thus providing an alternative solution that allows high temperature PIA to be conducted on Si substrates.

Despite resulting in a rougher surface, a higher temperature is preferred in favour of a higher dopant activation rate. Attributed to a smaller band gap, thus a shallower donor level (55 meV), the activation of n-type dopants in 3C-SiC is easier than in 4H-SiC (80–130 meV) [46]. Studies on n-type 3C-SiC suggest that nitrogen has advantages over phosphorous for use as an n-type dopant, with both fewer defects and lower resistivity achieved [42]. Compared with the N saturation density in 4H-SiC (around 5 × 10^19^ cm^−3^) [47], the level in 3C-SiC turns out to be similar at around 7 × 10^19^ cm^−3^ [48]. With the valence band aligned to other polytypes, the deep acceptor level issue still exists for 3C-SiC. Adding to the limited processing temperature, p-type implant and activation has long been an issue for 3C-SiC-on-Si [38,49]. In recent years, the developments on free standing 3C-SiC materials [50,51] make PIA temperatures above 1400 °C possible, thus facilitating a significant step forward in 3C-SiC power device fabrication. However, the knowledge of p-type 3C-SiC ion implantation and activation is very limited and requires further investigation. Table 3 summarises some past results published on the ion implantation and activation of dopants in 3C-SiC.

### 3.3. Ohmic Contact

Due to the requirement of an extra PIA process, achieving ohmic contacts on implanted regions is more difficult than on epilayers. As is the case in 4H-SiC [56], this is particularly true for p-type 3C-SiC because the acceptor levels are deeper, as previously mentioned. Attributed to a lower conduction band edge (3.8 eV from vacuum level), the theoretical SBH between 3C-SiC and commonly used metals is 0.9 eV lower than for 4H-SiC. This is convenient for n-type ohmic contact fabrication, while p-type remains as challenging as in other polytypes. Most work on SiC ohmic contacts is divided into three topics, namely surface preparation, contact metal, and post metallisation annealing (PMA).

The 3C-SiC epilayer surface roughness can vary significantly, from as low as 1 nm depending on the growth technique [43] to high values reaching tens of nm [57]. To achieve a relatively smooth semiconductor surface for ohmic contact fabrication, chemical mechanical polishing (CMP) is often used prior to any further processing. Noh et al. [58] show that the RMS surface roughness reduced from ≈20 nm to ≈7.5 nm. Consequently, the ohmic contact resistivity *ρ_c_* was reduced by an order of magnitude, from 8.6 × 10^−1^ Ωcm^2^ to 2.8 × 10^−2^ Ωcm^2^. As alluded to previously, practical device fabrication requires a high temperature (above 1400 °C) PIA treatment, which has been shown to degrade the surface following initial CMP. In [43], a detailed discussion was reported around the PIA effects on 3C-SiC surface morphology and its correlation to the resulting *ρ_c_* values. It was communicated that although severe damage to the surface can limit performance, the *ρ_c_* value will not be seriously affected given that the surface roughness value remains below 10 nm.

Many metals or metal stacks, including Al [54,59,60,61], Ti [54,59,60,61], Ni [37,54,57,58,60,61,62,63], Ni/Ti [43,55,61], Au/Ti [61], Pt [63], W [37], and TiW [64], have been analysed for 3C-SiC n-type ohmic contact fabrication. It was observed that Al contacts typically display the lowest *ρ_c_*, which was explained by the near-zero SBH between Al and 3C-SiC (∼0 eV) compared to Ti (0.4 eV) and Ni (0.55 eV) [54]. Nonetheless, both Ti and Al are readily oxidised in air, with Al characterised by a melting point below 600 °C. Conversely, Ni demonstrates a slow rate of oxidation at room temperature combined with a very high melting point. Although Ni reacts with SiC at temperatures higher than 500 °C, the Ni silicide microstructure helps to reduce the SBH. This in turn leads to a lower *ρ_c_*. Consequently, Ni is the most commonly utilised metal contact to n-type SiC.

**Figure 4:**
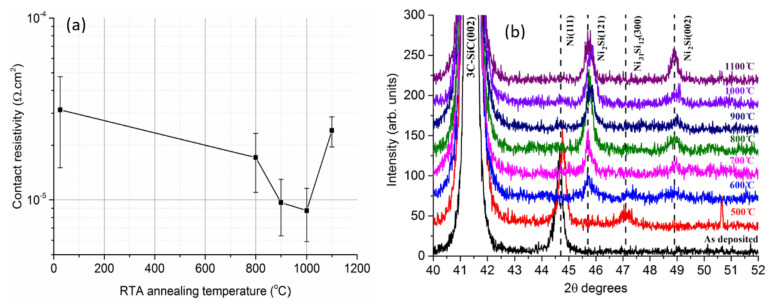
(**a**) Specific contact resistance dependence on the PMA temperature and, (**b**) XRD measurements of metal/3C-SiC (6 × 10^20^ cm^−3^) interface after various PMA temperatures indicating silicide formation. Contact was fabricated by depositing (Ti30 nm/Ni100 nm) on 5 × 10^20^ cm^−3^ N implanted 3C-SiC.

The effects of PMA on ohmic contacts fabricated on n-type implanted 3C-SiC (Figure 4a) shows a continuous reduction of contact resistivity with increasing annealing temperature up to 1000 °C, above which the resistance increases. Details of the silicide formation are shown by XRD analysis in Figure 4b. It can be inferred that between 500 °C and 600 °C, a coexistence of Ni_2_Si (121) and Ni_31_Si_12_ (300) is present. The Ni_31_Si_12_ (300) peak gradually diminishes at higher temperature, while the Ni_2_Si (002) becomes prominent and enhances continuously to temperatures reaching 1100 °C. Noting that Ni_2_Si (121) is readily formed at 600 °C, with no other noticeable phases above that temperature, the Ni_2_Si (002) enhanced phase could explain the contact resistance reduction from 800 °C to 1000 °C. It is worth mentioning that, due to the very low SBH of highly doped n-type 3C-SiC/metal interface, as-deposited ohmic contacts can be obtained without PMA processing [59,65]. This makes it possible to integrate SiC transistor technologies with other low temperature technologies, such as atomic layer deposited high k dielectrics (e.g., HfO_2_ or Al_2_O_3_) with relatively low growth temperatures and classic wafer bonded or heterojunction devices.

Compared to n-type 3C-SiC, even less is known about p-type 3C-SiC ohmic contacts. As with 4H-SiC, Al based alloys are most commonly used for p-type ohmic contact since very often Al is also the doping species. A Ti interlayer is often applied not only to improve the adhesion, but the TiC product after PMA also helps to reduce the contact resistance [36,66].

**Figure 5:**
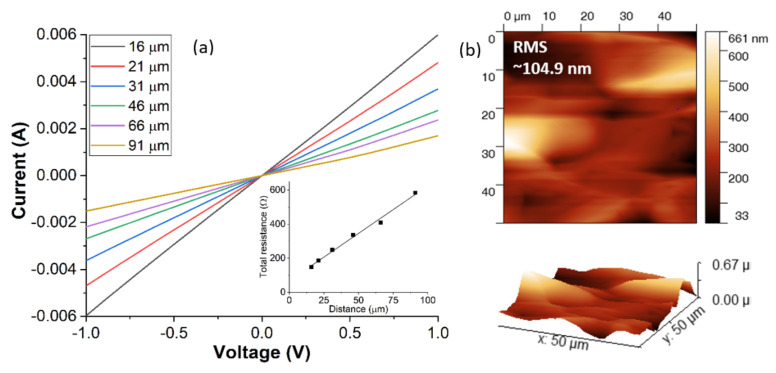
(**a**) I-V characteristic ohmic contact (Ni/Al/Ti) fabricated on Al implanted free standing 3C-Si and, (**b**) surface morphology of the free standing 3C-SiC by AFM.

Among the very limited data, the lowest specific contact resistances (10^−5^–10^−4^ Ωcm^2^) are obtained from trials made on p-type epilayers [66,67], which eliminates the issue of acceptor activation. However, when fabricating power devices such as MOSFETs, it is crucial to obtain ohmic contacts on selective highly doped, mostly implanted p+ regions. It is reported in [38] that, on 3C-SiC with a Si substrate, a hot implant (500 °C) with high Al concentration (1 × 10^20^ cm^−3^) together with very long duration (>300 h) PIA at 1300 °C had to be performed to achieve ohmic contacts, and even so, the resultant *ρ_c_* was still high, around the 10^−2^ Ωcm^2^ level. More recently, p-type ohmic contacts (Figure 5a) on Al hot implanted (600 °C, 1 × 10^20^ cm^−3^) free standing 3C-SiC have been reported. By increasing the PIA temperature treatment to 1700 °C, a dramatic reduction in annealing time was required—down to 2 h. Even though the *ρ_c_* value is still relatively high ~10^−3^ Ωcm^2^, it is promising since the contacts were fabricated on a very rough surface (Figure 5b), which can be further improved either by optimising the 3C-SiC growth process or additional polishing treatments. Table 4 provides a survey of the literature results for ohmic contact processing on 3C-SiC, mostly n-type.

### 3.4. MOS Processing

Given the superior electrical performance of SiC and its capacity to be thermally oxidised, it is not surprising that there are copious amounts of SiC MOS devices being demonstrated. The commercialised 4H-SiC polytype is naturally the most frequently reported. Numerous literature reports suggest that MOS interface traps are similar in nature for all SiC polytypes [69]. Therefore, studies relating to the 4H-SiC/SiO_2_ interface provide insightful information with respect to the equivalent 3C-SiC system.

**Figure 6:**
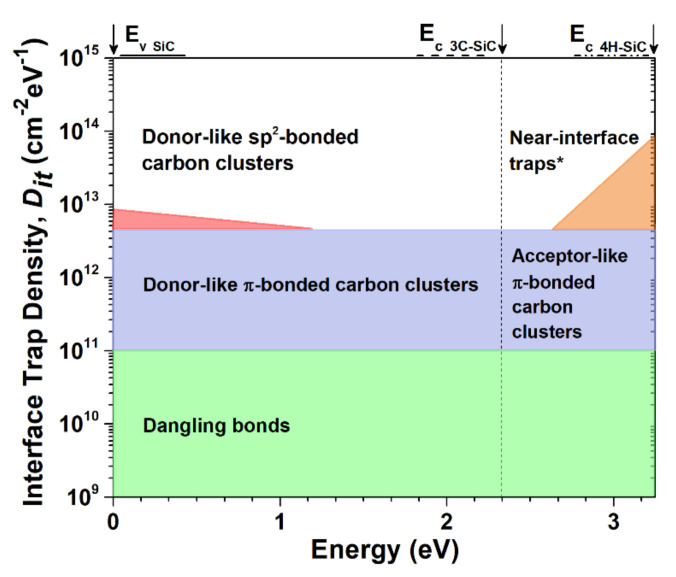
Schematic representation of the “carbon cluster model”. Adapted from Ref. [71] with permission from the author (R. Esteve).

In [70], reporting around the possible origins of interface traps identified two primary sources; namely, carbon and oxide defects that accumulate at the MOS interface during the oxidation process. The oxide defect-induced traps (also known as “near-interface traps”) have much smaller time constants compared to the carbon-clusters. Therefore, oxide defect-induced traps are also known as “fast traps” while the latter are coined “slow traps”. A graphic illustration of the carbon cluster model is shown in Figure 6, including the corresponding specified energy levels of the traps. Figure 6 shows the 4H-SiC conduction band edge is overwhelmingly impacted by π-bonded clusters and carbon near-interface traps, with the latter being most dominant. Both trap forms are acceptor-like, therefore negatively charged when occupied, which explains the positive flat band voltage (*V_fb_*) typically detected with respect to 4H-SiC MOS devices. In contrast, the 3C-SiC conduction band is devoid of near-interface traps due to a narrower band gap. However, 3C-SiC is still negatively-impacted by π-bonded carbon clusters. These (carbon clusters near the 3C-SiC conduction band edge) defects are positively charged if occupied as they are donorlike, resulting in a *V_fb_* that is negative. Dangling bonds augment the interface defectiveness but are negligible secondary concerns compared to the aforementioned carbon clusters. Consequently, hydrogen annealing is not as effective for SiC when compared to Si. Alternative methods have been demonstrated for high-quality SiC/SiO_2_ interface optimisation.

Reports focussing on the improvement of the SiC/SiO_2_ interface are mainly related to the topic of post oxidation annealing (POA). Former research literature revealed the advantages of including hydrogenation processes either during the (gate) oxidation process or subsequently, via the POA. This has the effect of decreasing the interface trap density (*D_it_*) in addition to reducing positive fixed charge (*Q_fc_*) [68,72]. Consequently, wet oxidation in conjunction with POA is often utilised for 3C-SiC MOSFET fabrication [73,74]. Regarding the nitridation step, extra deep interface traps revealed by double peak conductance spectra were observed from fabricated MOS capacitors via direct N_2_O oxidation and pure O_2_ oxidation methods on nitrogen implanted films [72,75].

**Figure 7:**
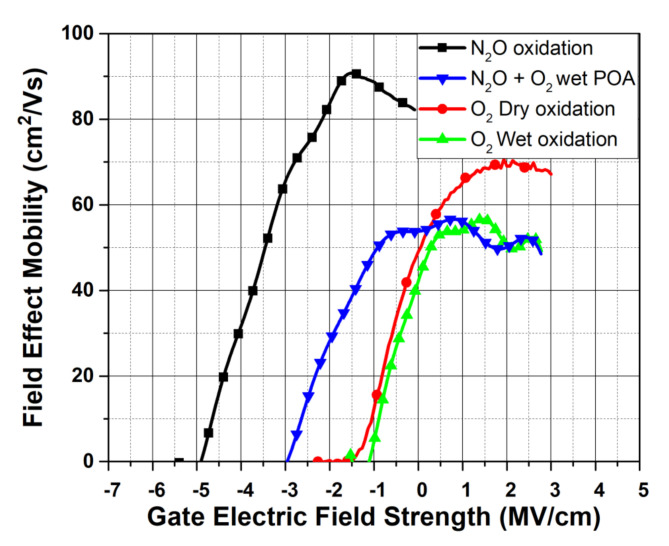
Transfer curves of 1300 °C oxidized lateral MOSFET with various conditions.

Figure 7 shows the lateral MOSFET transfer curves on Al implanted 3C-SiC/Si substrates with the gate oxide grown in different atmospheres, but all at the same temperature of 1300 °C [76]. Due to varying oxide thicknesses, for direct comparison the gate field instead of gate voltage is plotted on the x-axis. It can be inferred that the dry oxidized device demonstrates a normally on characteristic with a gate threshold voltage approaching zero. This is in agreement with the previously introduced Carbon Cluster Model, stating that only donor-like states occupy the 3C-SiC/SiO_2_ interface. Since these states are positively charged when vacant, these donor-like states may be responsible for the inherent negative threshold. The nitrided sample is even further shifted in the negative threshold direction due to the counter doping channel effect [77]. The wet oxidized sample has the most positive gate threshold. A combination of N_2_O nitridation and POA (wet) yielded an intermediate threshold field of around −2MV/cm. Clearly, the wet oxidation was successful in shifting the device threshold in a more positive manner, either by forcing a reduction in positive fixed oxide charges or via compensating them with additional negative charge. Both wet POA and oxidized processed devices have a peak field-effect mobility (*µ_FE_*) value in the region of 60 cm^2^/Vs, which is the lowest compared with the dry oxidized sample (70 cm^2^/Vs) and the N_2_O nitrided sample (90 cm^2^/Vs).

**Figure 8:**
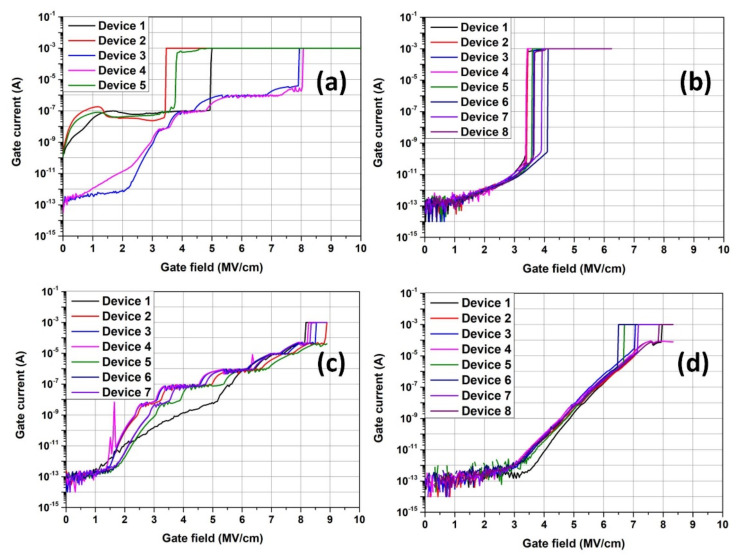
Dielectric breakdown curve of gate oxides fabricated by (**a**) 60 min 1300 °C O_2_ dry oxidation, (**b**) 15 min 1300 °C O_2_ wet oxidation, (**c**) 120 min 1300 °C N_2_O dry oxidation and (**d**) 30 min 1300 °C O_2_ dry oxidation + 90 min 1300 °C N_2_O POA.

As mentioned previously, the reliability of the 3C-SiC MOS system is particularly interesting, yet there has been relatively little study of this topic, mainly due to a shortage of non-defective 3C-SiC material. Figure 8 shows the critical strength (*E_c_*) of SiO_2_ layers grown on 3C-SiC/Si substrates in different atmospheres at 1300 °C. As can be seen, by using combined dry O_2_ gate oxidation with an N_2_O POA process, the noise level was greatly reduced and the critical electric field strength was able to be kept at around 8 MV/cm, the highest value observed.

**Figure 9:**
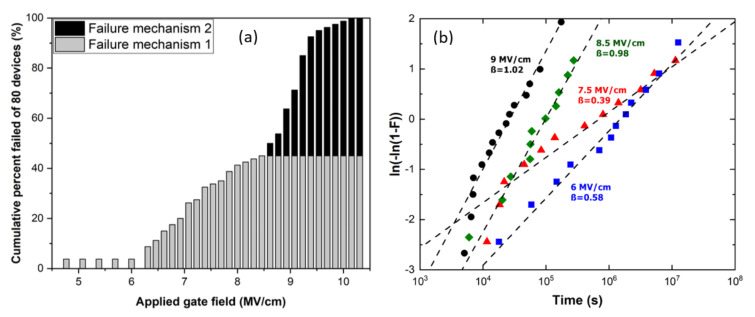
(**a**) Failure distribution of 3C-SiC MOS capacitors in the electric field range of 4.5–10.5 MV/cm, and (**b**) Weibull distributions of device failures at various electric fields.

Recently the reliability of 3C-SiC MOS capacitors (dry oxidised and N_2_O POA at 1300 °C) has been examined at room temperature by using both v-ramp and time-dependent dielectric breakdown (TDDB) analysis. As can be seen in Figure 9a, the accumulated total failure percentage increases steadily until around 8.5 MV/cm, beyond which the failure number sharply increases to 100%. The failures at lower fields are most likely induced by crystal deficiencies in the 3C-SiC substrate that alter localised material properties. High field (>8.5 MV/cm) failures are characterized by either F-N tunnelling, observed via the increased leakage current, or electron impact ionization energy being reached within the oxide due to elevated electric fields. TDDB analysis is conducted at electric field values of 6, 7.5, 8.5, and 9 MV/cm. The Weibull distributions are displayed in Figure 9b. Even at high fields beyond 8.5 MV/cm, the slope values remain low in the region of ~1, an order of magnitude lower than reported values for 4H-SiC [78], suggesting extrinsic defects are still the dominant failure mechanism.

**Figure 10:**
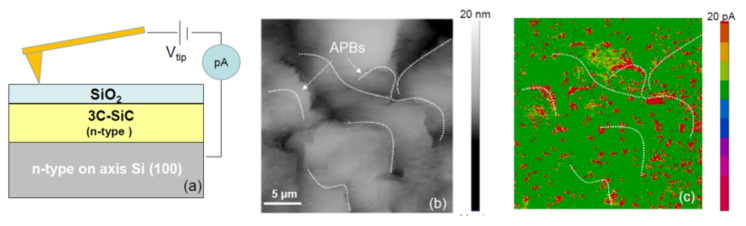
(**a**) C-AFM set-up adopted for the electrical characterization of the SiO_2_/3C-SiC system; (**b**) AFM morphology and (**c**) C-AFM current map acquired under the application of an electric field of 8 MV/cm to the tip. Adapted with permission from ref. [79]. Copyright © 2021 Elsevier Ltd.

Besides the application on Schottky contacts described in Section 3.1, nanoscale resolution current mapping by C-AFM can also be a powerful analysis technique for investigation of the dielectric breakdown behaviour of thin insulators. In fact, this method was recently employed by Fiorenza et al. [79] in order to explain the reasons behind the premature breakdown of thermal oxide (SiO_2_) grown on 3C-SiC typically observed in MOS capacitors, by stressing the oxide through the application of a bias to the C-AFM tip corresponding to an electric field of 8 MV/cm (see schematic set-up in Figure 10a).

The C-AFM current map and corresponding AFM surface morphology acquired on the SiO_2_/SiC system are reported in Figure 10b,c, respectively. The C-AFM current map in Figure 10c reflects the breakdown distribution of an array of tip/oxide nano-MOS capacitors. Hence, the features on the surface morphology (Figure 10b) could be correlated with the position of the breakdown spots (Figure 10c), which are not randomly distributed, but preferentially appear along the APBs (dashed line in Figure 10b). Here, the straight line conductive aspects associated with SFs on the exposed 3C-SiC surface (see Figure 10c) were not visible in the presence of a thermal oxide. Based on this analysis, the premature dielectric breakdown observed in MOS capacitors could be attributed to the presence of positively charged APBs, causing an electron injection enhancement from the 3C-SiC into the SiO_2_.

Table 5 is a list of recent work performed on the study of 3C-SiC MOS interface traps.

## 4. 3C-SiC Device Prototypes

### 4.1. Schottky Diode

The study of Schottky and p-n junction diode behaviour on thin film CVD 3C-SiC dates back to the 1980s [83]. Much of this early work was conducted on thin films deposited on silicon and 6H-SiC. The initial studies were concerned with the surface science of fabricating appropriate metal contacts. These diodes demonstrated the first reported 3C-SiC rectification behaviour of up to 200 V, with leakage currents ranging from between 10^−4^–1 A/cm^2^ [84,85]. Vertical heterojunction Schottky diodes based on platinum (Pt) contacts showed a blocking voltage of 85 V with a low forward voltage drop of ~0.85 V [86]. Gold contacts to 3C-SiC for Schottky diode applications displayed a variance of the barrier height with contact area [19]. This can be explained by the defect density inherent within the starting material. More recent Schottky diode reports suggest that the leakage current is not dominated by SF density, as the leakage current had a greater dependency on the barrier height [87]. Barrier height nonuniformities of the Schottky barrier have been observed on lateral 3C-SiC-on-Si diodes, implicating complex trapping/de-trapping phenomena observed within the material [88]. The information acquired has led to validated technology computer aided design (TCAD) models for accurate 3C-SiC device simulation [9].

### 4.2. PiN Diode

Attributed to its smaller bandgap, 3C-SiC has a lower p-n junction built-in potential (≈1.75 V) than 4H-SiC (≈3 V). In [9] it is shown that, up to 4.5 kV blocking voltage, the forward voltage drop at 250 A/cm^2^ remains lower for 3C-SiC than 4H-SiC in PiN diode applications. Until recently, however, fabricating 3C-SiC PiN diodes has been difficult, not only because of the high defect density within 3C-SiC epilayers caused by the lattice mismatch with Si [21], but also due to the lateral nature of structures necessary to avoid the 3C-SiC/Si heterojunction. While there are several reports on achieving n-type conduction in 3C-SiC epi/implanted layers [42,48,66], and p-type conduction in Al doped epilayers [66,89], it remains an obstacle for p-type implanted layers. This is mainly due to the post implantation anneal temperature, which was limited to the Si melting point, 1414 °C, which is not sufficient to activate the deep level Al dopants, even if hot implantation was applied.

**Figure 11:**
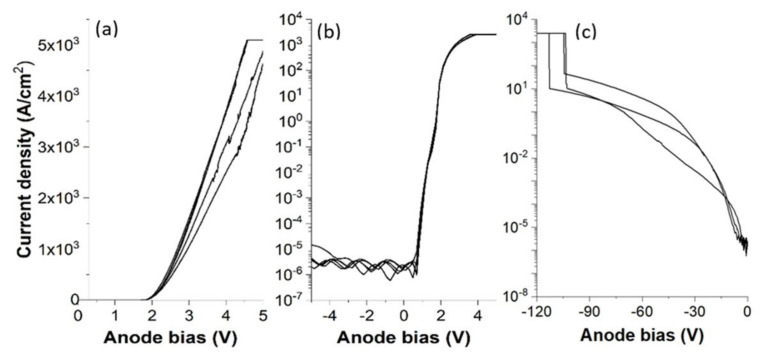
(**a**) Forward J-V characteristics, (**b**) on-off performance at ±5 V, and (**c**) reverse breakdown of bulk 3C-SiC PiN diodes.

Low voltage lateral p-n junction diodes were previously demonstrated via the formation of implanted n+ regions in p-type doped 3C-SiC epilayers grown on Si substrates [90,91]. However, to make the most of its benefits in power applications, a vertical structure is necessary. 3C-SiC growth methods have improved in recent years [6,92,93], and bulk 3C-SiC are now available [51]; thus, a higher annealing temperature can now be applied. Vertical PiN diodes were fabricated on free standing 3C-SiC material by implanting Al in n-type doped epilayer and the forward current density is shown in Figure 11a. The built-in potential of the fabricated PiN diode is around 2 V, slightly higher than the theoretical value 1.75 V [9], but it is still much lower than the typical >3 V for 4H-SiC [94,95,96]. The forward current density goes above 1000 A/cm^2^ at 2.7 V, and the lowest differential resistance is estimated to be 0.5 mΩcm^2^. The device on–off ratio at ±5 V is as high as 10^9^, as shown in Figure 11b, and a blocking voltage above 100 V is achieved (Figure 11c). An observation to note with respect to bipolar PiN diode I-V characterisation is that no bipolar degradation has been reported in the literature with respect to 3C-SiC pn diodes. This is most likely due to the fact that attention is being placed upon more fundamental device limiting issues such as SF-induced leakage currents.

### 4.3. MOSFET

Early 3C-SiC power devices were predominantly demonstrated via heteroepitaxial 3C-SiC grown by chemical vapour deposition (CVD) above silicon substrates in addition to free-standing wafers, provided by HOYA Advanced Semiconductor Technologies Co Ltd. [50,74,97,98]. Power devices were based on diode and MOSFET (lateral and vertical) architectures. Devices demonstrated by 3C-SiC CVD grown on undulant-silicon substrates suffered from premature breakdown voltage and high leakage currents due to APBs and SF inherent within the epitaxial layer of the device [99,100].

Typical characteristics showed that achieving breakdown voltages in excess of 600 V was challenging since the leakage current emanating from the formerly mentioned p-n junction SFs degraded performance in a terminal manner [100]. High current cellular vertical 3C-SiC MOSFETs were demonstrated by Abe et al. [74]. This device achieved an impressive 1220 A/cm^2^ current density based on a single cell. This corresponds to a current carrying capability of 41–132 A for a 3 × 3 mm^2^, 600 V chip. SF-induced leakage current hampered the off-state performance of this MOSFET. CVD deposited gates produced 600 V-MOSFETs with a high channel mobility of 200 cm^2^/Vs [101]. The high channel mobility and low specific on-state resistance of 5–7 mΩcm^2^ were brought about by a specific activation anneal of 1600 °C in argon (Ar), in order to realise a smooth 3C-SiC surface prior to deposition of the gate oxide. They used 600 V DMOSFETs to show that material quality has a strong influence on the blocking behaviour. In contrast, the on-state electrical characteristics were unaffected [102]. A 200V reduction in breakdown voltage was observed for DMOSFETs with a high crystal defect density.

Due to the lower interface trap density at the 3C-SiC/SiO_2_ interface compared with 4H-SiC, MOSFETs are the most studied 3C-SiC devices, targeting for lower on-resistance than 4H-SiC MOSFETs in medium voltage applications (600–1200V). High field-effect mobility values were demonstrated by fabricating 3C-SiC MOSFETs with a high current density of 1220 A/cm^2^ and encouraging scaling features were shown in 1 mm × 1 mm and 3 mm × 3 mm devices [74]. In addition, it is shown in [65,68] that by removing the rapid thermal anneal for the ohmic contact, the field-effect mobility can be further improved. Despite the achievements made in forward conditions, reaching blocking ability (BV) close to the theoretical values is still a challenge, mainly because of the high leakage current induced by crystal defects such as SFs [97]. By reducing stacking faults to ~90 cm^−1^, the device blocking ability (5 × 10^15^ cm^−3^ doped drift region) can be significantly improved to 600 V [50], close to the unipolar limit. Table 6 is a summary of the recent literature results for 3C-SiC MOSFET fabrication.

## 5. Conclusions

This paper gave an overview of the processing technology associated with heteroepitaxial 3C-SiC-on-silicon, including the more recently available bulk 3C-SiC studies. This topic is highly relevant today since this material presents some clear advantages over its commercial WBG competitors in terms of MOS channel resistance and reliability. These factors are of the utmost importance when considering that it is the automotive sector that is driving the widespread uptake of WBG technologies. Schottky contact processing on 3C-SiC has mainly been conducted on heteroepitaxy (on-silicon) utilising high work function metals such as Au or Pt. These rectifying contacts are typically characterised by high leakage currents arising from SFs and APBs and it is clear that a step-change in material quality is needed for power device applications. To the best of the authors’ knowledge, there remains no semiconductor device grade wafer supplier of bulk 3C-SiC. However, heteroepitaxial 3C-SiC-on-silicon is available up to a wafer diameter of 4 inch. The main obstacle to large diameter 3C-SiC commercialisation remains the SF density that ranges from 200–5000 cm^−1^. Hence, the future prospects for 3C-SiC are incumbent upon reducing SFs and APBs, which remains key to realising large diameter 3C-SiC bulk wafer production. 3C-SiC-on-silicon demonstrates serious limitations when the ion implantation process is taken into consideration. Therefore, the majority of studies to date have used conventional PIA annealing up to 1400 °C (melting temperature of silicon substrate) and pulsed laser annealing. Generally, dopant activation rates are low in 3C-SiC heteroepitaxy structures, although recently more promising behaviour has been described on free standing (bulk) 3C-SiC. Most recently p-type aluminium doped 3C-SiC has been demonstrated with weak p-type behaviour. N-type ohmic contacts have been consistently achieved using metals such as Ni, Al, Ti, Au and W demonstrating specific contact resistivities as low as 5 × 10^−7^ Ωcm^2^. The success is related to the high n-type ion implantation activation/ionisation rates accompanied by the low donor levels relative to 4H-SiC. P-type ohmicity based on metals including Al, Ni, Ti and poly-silicon have produced resistances in the region of ~10^−5^ Ωcm^2^. Compared to n-type donor levels in 3C-SiC, p-type acceptor energy levels are closer to the midgap, resulting in a lower degree of acceptor ionization. Diodes based on Schottky and PiN designs have been demonstrated on 3C-SiC. The state of the art with respect to diodes are bulk PiN structures with a built-in voltage of 2V and current density of 1000 Acm^−2^ observed. The 3C-SiC MOS interface is relatively untroubled by near interface traps when compared to its 4H-SiC counterpart. This can be inferred from experimental results based on nitrogen anneals where channel mobilities approaching 100 cm^2^/Vs have been observed. Again nitrogen-based thermal oxidation produced interface trap densities in the region of 10^11^ cm^−2^ eV^−1^. A reliability analysis of the 3C-SiC MOS interface revealed high breakdown fields in the region of 8MV/cm including cumulative device failure arising primarily from 3C-SiC crystal defects (TDDB). Actual MOSFET demonstrators are plagued by high leakage currents resulting from crystal defects. Thus, 600V 3C-SiC MOSFETs that approach the theoretical unipolar limit have been demonstrated.

## Figures and Tables

**Table 1:** Appropriate physical and electrical properties of cubic silicon carbide (3C-SiC) compared to other wide bandgap materials (data taken at 300 K).

Material	Band Gap, (eV)	Intrinsic Carrier Conc., (cm^−3^)	Dielectric Constant	Electron Mobility (cm^2^/Vs)	Critical Electric Field (MV/cm)	Saturation Velocity (10^7^ cm/s)	Thermal Conductivity (W/cmK)	Baliga Figure of Merit
Si	1.12	1.5 × 10^10^	11.8	1350	0.2	1.0	1.5	1
GaAs	1.42	1.8 × 10^6^	13.1	8500	0.4	1.2	0.55	29
3C-SiC	2.36	1.5 × 10^−1^	9.7	800	1.4	2.5	3.2	86
4H-SiC	3.26	8.2 × 10^−9^	10	720 ^a^ 650 ^c^	2.8	2.0	4.5	556
2H-GaN	3.39	1.9 × 10^−10^	9.9	1000 ^a^ 2000 **	3.75 ^a^ 3.3 *	2.5	1.3	3175
Ga_2_O_3_	4.85	2.6 × 10^−9^ −1.0 × 10^−22^	10	300	8	1.8–2.0	0.1–0.3	6171
Diamond	5.45	1.6 × 10^−27^	5.5	3800	10	2.7	22	8.4 × 10^4^
2H-AlN	6.2	10^−34^	8.5	300	12 *	1.7	2.85	1.8 × 10^4^

Note: ^a^ is mobility along a-axis, ^c^ is mobility along c-axis, * refers to an estimated value and ** refers to the 2DEG mobility.

**Table 3:** A summary of literature data on the ion implantation and activation of 3C-SiC.

Material	Implantation	PIA	Activation Rate	Ref.
**N-Type**				
2 × 10^17^ cm^−3^ p-type 3C-SiC(100)/Si	RT ^1^, N, peak 5 × 10^19^/5 × 10^20^ cm^−3^	None	0.44%/0.55%	[52]
	400 °C, N, peak 5 × 10^19^ cm^−3^		1.35%	
	800 °C, N, peak 5 × 10^19^/5 × 10^20^cm^−3^		15%/50.8%	
1 × 10^18^ cm^−3^ p-type 3C-SiC(100)/Si	800 °C, N, peak 5 × 10^19^ cm^−3^	None	12.4%	[53]
	900 °C, N, peak 5 × 10^19^ cm^−3^		14.8%	
	1000 °C, N, peak 5 × 10^19^ cm^−3^		18.4%	
	1100 °C, N, peak 5 × 10^19^ cm^−3^		36.0%	
	1200 °C, N, peak 5 × 10^19^ cm^−3^		52.2%	
1 × 10^16^ cm^−3^ p-type 3C-SiC(100)	RT, N, peak 1 × 10^20^ cm^−3^	10 min in Ar at 1500 °C	68%	[49]
1 × 10^16^ cm^−3^ p-type 3C-SiC(100)	RT, N, peak 6 × 10^19^ cm^−3^	10 min in Ar at 1400 °C	80%	[54]
<1 × 10^16^ cm^−3^ n-type 3C-SiC(100)/Si	RT, N, peak 5 × 10^20^ cm^−3^	1 h in Ar at 1150 °C	6.5%	[46]
		1 h in Ar at 1350 °C	13%	
<1 × 10^16^ cm^−3^ n-type 3C-SiC(100)/Si	RT, N, peak 5 × 10^19^ cm^−3^	1 h in Ar at 1150 °C	40%	[44]
		1 h in Ar at 1350 °C	57%	
		1 h in Ar at 1400 °C	100%	
<1 × 10^16^ cm^−3^ n-type 3C-SiC(100/Si	RT, N, peak 5 × 10^19^/5 × 10^20^ cm^−3^	1 h in Ar at 1350 °C	60%/17%	[55]
<1 × 10^16^ cm^−3^ n-type 3C-SiC(100/Si	RT, N, peak 1.5 × 10^19^/6 × 10^20^ cm^−3^	1 h in Ar at 1375 °C	100%/12%	[49]
**P-type**				
2.8 × 10^16^ cm^−3^ n-type 3C-SiC(100)/Si	RT and 850 °C, Al and B, peak 5 × 10^19^–1 × 10^20^ cm^−3^	10 min in N_2_ at 1200 °C	Too low, n-type behaviour	[50]
<1 × 10^16^ cm^−3^ n-type 3C-SiC(100)/Si	500 °C, Al, peak 1 × 10^20^ cm^−3^	317–546 h in Ar at 1300 °C	Weak p-type behaviour	[38]

^1^ Room temperature.

**Table 4:** A summary of literature data on the fabrication of 3C-SiC ohmic contact.

Contact	Doping (cm^−3^)	PMA Conditions	*ρ_c_* (Ωcm^2^)	Ref.
**N-Type**				
Al	5 × 10^18^ N implanted	As-deposited	1 × 10^−4^	[59]
	3 × 10^19^ N implanted		6 × 10^−5^	
	1 × 10^20^ N implanted		5 × 10^−5^	
	3 × 10^20^ N implanted		1.3 × 10^−5^	
	6 × 10^19^ N implanted	As-deposited/500 °C	5 × 10^−7^/6 × 10^−5^	[54]
	6 × 10^18^ N implanted	300 °C	5 × 10^−7^	[61]
	1 × 10^17^ N doped epi	As deposited/500 °C	2 × 10^−4^/1 × 10^−4^	[37]
Ti	5 × 10^18^ N implanted	As-deposited	7 × 10^−5^	[59]
	3 × 10^19^ N implanted		4 × 10^−5^	
	1 × 10^20^ N implanted		2 × 10^−5^	
	3 × 10^20^ N implanted		1.5 × 10^−5^	
	6 × 10^19^ N implanted	As-deposited/500 °C	5 × 10^−6^/6 × 10^−5^	[54]
Ni	Not known, N doped epi	1000°C	3.7 × 10^−4^	[58]
	6 × 10^19^ N implanted	As-deposited/500 °C	2 × 10^−5^/5 × 10^−6^	[54]
	3 × 10^19^ N doped epi	950 °C	1.2 × 10^−5^	[61]
	1 × 10^17^ N doped epi	As-deposited/500 °C	5 × 10^−4^/5 × 10^−5^	[37]
	1 × 10^20^ P implanted	1000 °C	1.4 × 10^−5^	[61]
	Not known, poly crystal epi	As-deposited	1.6 × 10^−6^	[65]
	5 × 10^17^ N doped epi	950 °C	1.5 × 10^−5^	[57]
	1 × 10^17^ N doped epi	950 °C	3.7 × 10^−3^	[66]
Ni/Ti	5 × 10^19^ N implanted	As-deposited	7 × 10^−4^	[65]
	5 × 10^20^ N implanted		3 × 10^−5^	
	1 × 10^19^ N implanted	1000 °C	2 × 10^−4^	
	5 × 10^19^ N implanted		4 × 10^−5^	
	5 × 10^20^ N implanted		9 × 10^−6^	
	5 × 10^20^ N implanted	1000 °C	8 × 10^−6^	[43]
	5 × 10^20^ P implanted		2 × 10^−5^	
	>10^20^ N implanted	1050 °C	2 × 10^−5^	[61]
	5 × 10^19^ N implanted	1000 °C	3.2 × 10^−6^	[55]
Au/Ti	3 × 10^20^ N implanted	600 °C	1.2 × 10^−5^	[61]
Pt	Not know, poly crystal N doped epi	As-deposited	1.2 × 10^−5^	[63]
W	1 × 10^17^ N doped epi	As-deposited/500 °C	2 × 10^−3^/2 × 10^−3^	[37]
TiW	Not know, N doped epi	1000 °C	4.6 × 10^−4^	[64]
	4 × 10^19^ N/P implanted	As-deposited	ohmic	[68]
**P-type**				
Al	1.33 × 10^17^ Al doped epi	710 °C	1.4 × 10^−2^	[67]
Al/Poly			3.5 × 10^−4^	
Ni/Al/Ti	5 × 10^19^ Al doped epi	950 °C	1.8 × 10^−5^	[66]
	1 × 10^20^ Al implanted	1000 °C	10^−2^	[38]
	1 × 10^20^ Al implanted	1000 °C	10^−3^	This work

**Table 5:** A summary of literature data on the processing of 3C-SiC MOS interface and relevant information on fixed charges (*Q_fc_*), interface trap density (*D_it_*), and oxide critical field (*E_c_*), unless specified, the 3C-SiC materials listed are epilayers.

Oxidation Substrate	Oxidation	POA	*Q_fc_* (cm^−2^)	*D_it_* (cm^−2^ eV^−1^)	*E_c_* (MV/cm)	Ref.
n-type 3C-SiC	NO, 1175 °C, 4 h	-	-	~10^11^	-	[68]
Al implanted 3C-SiC	Dry O_2_, 1100 °C, 1.5 h	Wet O_2_, 950 °C, 3 h	-	5 × 10^12^–1 × 10^13^	-	[74]
n-type 3C-SiC	Dry O_2_, 1120 °C, 0.5 h	Ar, 1120 °C, 1 h	-	~10^12^	-	[75]
n-type 3C-SiC	PECVD (SiH_4_ + N_2_O)	N_2_O, 1100 °C, 3 h	2.01 × 10^12^	~10^12^	8.2	[80]
		Wet O_2_, 950 °C, 3 h	1.7 × 10^11^	~2 × 10^12^	9.1	
		Dry O_2_, 950 °C, 3 h	1.76 × 10^11^	~2 × 10^13^	5.9	
		N_2_, 1100 °C, 3 h	4.65 × 10^12^	~7 × 10^12^	6.3	
		N_2_, 950 °C, 3 h	2.63 × 10^12^	~2 × 10^13^	6.2	
n-type 3C-SiC	NO, 1185 °C, 2 h	-	-	~10^12^	-	[77]
	N_2_O, 1185 °C, 1 h	-	-	~8 × 10^11^	-	
n-type 3C-SiC	Dry O_2_, 1100 °C, 4 h	-	9.3 × 10^12^	4.27 × 10^13^	-	[73]
	Dry O_2_, 1200 °C, 1 h	-	7.1 × 10^12^	6.59 × 10^13^	-	
	Dry O_2_, 1100 °C, 1.5 h	O_2_, 950 °C, 3 h	1.3 × 10^12^	7.1 × 10^12^	-	
	Dry O_2_, 1100 °C, 1.5 h	Wet O_2_, 950 °C, 3 h	0.9 × 10^12^	5.2 × 10^12^	-	
	N_2_O, 1200 °C, 2 h	-	3.0 × 10^12^	1.15 × 10^13^	-	
	N_2_O, 1250 °C, 2 h		3.1 × 10^12^	9.1 × 10^12^	-	
	N_2_O, 1250 °C, 2 h	Wet O_2_, 950 °C, 3 h	1.6 × 10^12^	9.4 × 10^12^	-	
n-type 3C-SiC	PECVD (SiH_4_ + N_2_O)	N_2_, 950 °C	5.7–7 × 10^12^	5 × 10^11^–7 × 10^12^	-	[81]
n-type 3C-SiC	Dry O_2_, 1200 °C	-	1.1 × 10^12^	~10^12^	-	[82]
	Dry O_2_, 1300 °C		1.1 × 10^12^			
	Dry O_2_, 1400 °C		4.1 × 10^12^			

**Table 6:** A summary of literature data on the forward and reverse performance of 3C-SiC MOSFETs.

Structure	Channel	Oxidation	POA	*µ_FE_* (cm^2^/V.s)	*B_V_* (V)	Ref.
Lateral	2 × 10^17^ cm^−3^ p-type epi	Wet O_2_, 1150 °C, 2.5 h	Ar, 1150 °C, 0.5 h + Wet O_2_, 950 °C, 2 h	≈165	-	[73]
Lateral	1 × 10^16^ cm^−3^ p-type epi	Wet O_2_, 1100 °C	Ar, 1150 °C, 0.5 h + Wet O_2_, 800 °C, 0.5 h	≈229	-	[103]
Lateral	1 × 10^18^ cm^−3^ Al implanted	Dry O_2_, 1300 °C	-	≈80	-	[65]
Vertical	1 × 10^18^ cm^−3^ Al implanted	Dry O_2_, 1100 °C, 1.5h	Wet O_2_, 950 °C, 3 h	≈28	≈100	[68]
Vertical	1 × 10^18^ cm^−3^ Al implanted	Dry O_2_, 1100 °C, 1.5 h	Wet O_2_, 950 °C, 3 h	≈45	550–600	[50]
Vertical	Al implanted	Wet O_2_, 1150 °C,	-	>100		[104]

## Data Availability

The data underlying this article will be shared on reasonable request from the corresponding author.
